# Factors associated with the depression among people with disabilities: A cross-sectional study in Chinese communities of Shanghai

**DOI:** 10.1097/MD.0000000000023331

**Published:** 2020-11-20

**Authors:** Yahong Bi, Xincai Zhao, Yanyan Zhou, Limin Lao, Sunfang Jiang

**Affiliations:** a General Practice Department, Zhongshan Hospital, Fudan University, Shanghai; b Jiangsu Provincial People's Hospital, Nanjing, Jiangsu Province; c Department of Pharmacy, Shanghai Jiao Tong University Affiliated Sixth People's Hospital, Shanghai; d Xinzhuang Community Health Center, Minhang District, Shanghai; e Healthcare Center, Zhongshan Hospital, Fudan University, Shanghai, China.

**Keywords:** Chinese communities, depression, disabilities, factors

## Abstract

Depression has become a growing health issue in the world and is projected to become a leading cause of global burden. However, there is little scientific research on the factors associated with depression in people with disabilities in China. In this cross-sectional study, we aimed to explore the prevalence and related factors of depression among people with disabilities in communities in mainland China.

Participants with disability certificates were recruited via face-to-face interviews to complete questionnaires. Contents include participants’ demographic characteristics, the Modified Barthel Index (MBI), chronic medical history, and the Patient Health Questionnaire-9 (PHQ-9).

A total of 1815 participants (M age = 60.35 ± 13.66) whose questionnaires are eligible were finally included. Among them the incidence rate of depressive symptoms was up to 39.9%. Multifactor regression analysis showed that grade I disability (odds ratio (OR) = 1.37, *P* < .05), impairment activities of daily living (OR = 3.23, *P* < .001), diabetes (OR = 1.43, *P* < .05), and hyperlipidemia (OR = 1.59, *P* < .001) were associated with depression in the disabled. However, intelligence disability is a protective factor of depression (OR = 0.69, *P* < .05).

The data demonstrates that the depression of the disabled should arouse the attention of our society. Furthermore, the interventions to disability degree, impairment activities of daily living, diabetes, and hyperlipidemia may help to improve the mental health of the disabled people.

## 1 Introduction

Individuals with disabilities tend to be at high risk for mental health. Compared with the non-disabled, people with disabilities are always living in poor condition and need special care, and who have higher rate of poverty and fewer social contacts.^[1]^ The United Nations and the World Health Organization (WHO) have used the “International Classification of Functioning, Disability and Health, ICF” as the criteria for disability surveys and statistics. According to the ICF, the categories of the disability include visual, hearing, speaking, limbs, intelligence, mental, and multiple disorders. With the development of the “bio-psycho-social” model of the disabled people, mental health problems of them have aroused the worldwide concern.

Within the past decades, depression has become a growing health issue in the world and is projected to become a leading cause of global burden.^[2]^ According to the study of World Health Organization, depression is estimated to affect 350 million people in the world and will lasts for many years.^[3]^ Depression increases risk of suicide and has important implications for the onset and progression of other health problems, which often adversely affects individual's quality of life.^[4–7]^ When it comes to mortality, depression ranked ninth behind health problems killers such as heart disease, stroke, and HIV.^[3]^ Yet depression is generally lower in diagnosis and treatment because of disgrace, lack of effective therapies, and insufficient resources for mental health counseling.

Previous research suggests that depressive symptoms and anxiety are common mental health problems.^[8]^ But depression may develop when depressive symptoms and anxiety become severe and persistent.^[9]^ Depression is characterized by sadness, loss of interest in activities and by reduced energy. The extent of its severity, symptoms, and the duration of the disorder are quite different from normal physical mood changes (WHO, 2011). Besides, previous studies revealed that depression has high morbidity in low-income female,^[10]^ older people,^[11]^ and (ex-)military personnel with a physical impairment.^[12]^ And depression is also considered as one of the more prevalent issues in disabled persons. What's more, previous studies have found that the economic impact of disability is substantial among many factors. Multiple studies have shown a relationship between disability and depression.^[13–15]^ A confirmed diagnosis of people with severe depressive symptoms accounts for 8.2% of the total worldwide disabled persons.^[9]^ However, the incidence and related factors of depression among disabilities in communities have not been reported in China.

In 2006, the second national survey of disabled persons of China showed that the disabled persons accounted for 6.34% of the total population.^[16]^ As a vulnerable community in society, mental health of the disabled cannot be ignored. However, there is little scientific research on the factors associated with depression in people with disabilities in China. Therefore, the purpose of this study is to explore the depression condition and possible related factors of the disabled people in Jing’an District of Shanghai. Our study was based on a questionnaire survey which used the Modified Barthel Index (MBI) and the Patient Health Questionnaire-9 (PHQ-9) as a criterion.

## 2 Methods

### 2.1 Patients and study design

We performed a cross-sectional study using patients’ medical records and conducted at 5 streets in Jing’an district of Shanghai, China. This study was approved by Ethics Committee of Zhongshan Hospital. Informed consent was obtained from all individual participants through signing of informed consent forms.

Participants included were those who were identified as disabled. All people identified as disability either had a disability certificate or had a diagnosis of disability in their health records were recruited. A face-to-face questionnaire was conducted for the certified disabled persons from September 2016 to December 2017. All participants in the study needed to

(1) disabled persons with “disability certificates of People's Republic of China” or had a diagnosis of disability in their health records,
(2) be able to report their own functional status after interpretation, and
(3) volunteer to participate in this study.

The category and grade of disability were defined according to the disability certificate and health record. The data of chronic diseases such as hypertension and diabetes were obtained according to the hospital's health records. Disabled people with extremely severe disease, cognitive impairment, or other problems that are unable to cooperate with investigators would be excluded.

### 2.2 Instrumentation

This study mainly selected 2 scales:

(a) the MBI,
(b) the PHQ-9.

In total, the 2 questionnaires totaled 19 questions.

The participants’ activities of daily living (ADL) are assessed using the MBI. The Barthel Index, developed by Mshoney and Banhel in 1965, is one of the most widely used instrument by both researchers and clinicians in the field of rehabilitation.^[17]^ This instrument includes 10 items: feeding, personal hygiene, bathing, dressing, bowel control, bladder control, toilet transfer, bed/chair transfer, ambulation, and stair climbing. In 1989, Canadian scholars modified the Barthel Index on the basis of the original content to optimize the defects of coarse classification and low sensitivity. With good reliability, validity, and sensitivity, MBI can be more sensitive to the changes of the patient's activities of daily living.^[18,19]^ The Chinese version of the MBI was used in Hong Kong hospitals in the late 1990s, which has a better retest reliability.^[20]^

Over the past decade, various kinds of psychometric instruments are available for the diagnosis of depression, ranging from screening measures to more comprehensive self-report inventories. Of the many available screening instruments, the depression module of the PHQ-9 has been verified as a most sensitive instrument for many specific rehabilitation populations.^[21]^ The PHQ-9 was primarily developed by Spitzer according to the “Diagnostic and statistical manual of mental disorders fourth edition, DSM-IV” in 1999.^[22]^ The PHQ-9 items are based on the diagnostic criteria for depression, is a simple, time-saving, cost-effective, and easily scored method that is acceptable to participants and can be used to both screen for depression and assess depression severity.^[23]^ It rates the frequency of symptoms (from not at all to nearly every day) over the past 2 weeks on a scale from 0 to 3, with total scores ranging from 0 to 27. Because of its simplicity and ease of operation, PHQ-9 has been used as one of the preference tools to screen for depression in primary health centre.

### 2.3 Data collection procedures

After consent was obtained, face-to-face interviews were conducted by our researchers. Contents include participants’ demographic character, activities of daily living, chronic conditions, and mental health status, measured using the MBI and PHQ-9 scales. After the completion of the questionnaire, the investigator checked the completeness of the questionnaire and made a confirmation with participant in time to reduce the unqualified rate of the questionnaire. After completion of the questionnaire, all of them are collected. Furthermore, the completed questionnaires would be input and analyzed immediately.

### 2.4 Data analysis

Data collation, coding, and entry were conducted first using Excel2013 and Epidata3.1. Data analyses were performed using the statistics system SPSS 21.0. And chi-square test and logistic regression analysis were used for the description and analysis of prevalence and related factors of depression among people with disabilities. The MBI and PHQ-9 scores were based on the summation of each item. MBI, with total of 10 items, each item score 0 to 15, a total score of 100, 100 means completely self-independent. PHQ-9's standard: 0 to 4 points, no depression; 5 to 9 points, maybe slight depression; 10 to 14 points, may have moderate depression; 15 to 19 points, may have moderate–severe depressive disorder; 20 to 27 points, may have severe depression. The cutoff point of the PHQ-9 scale is defined as 5 scores. The incidence rate of the depression is the percentage of disabled people with the total scores of PHQ-9 scale more than 5.

## 3 Results

### 3.1 The general information of participants

1831 people with disability certificates enrolled in the survey and completed the questionnaires. 16 questionnaires were found to have missing “category of disability,” and were no longer included in the data analysis. Therefore, a total of 1815 participants completed usable questionnaires and were included in the last analyses. 1815 respondents included male 941 (51.96%) and female 870 (48.04%). The mean age of the sample was 60.35 ± 13.66 years, the maximum age is 97, and the minimum age is 7. All of them, there were 433 people with visual impairment (23.86%), 222 hearing disabled persons (12.23%), 20 people with speaking impairment (1.10%), 742 limbs handicapped (40.88%), 169 intellectual disability (9.31%), 198 mental deficiency (10.91%), and 31 people with multiple disabilities (1.71%), as indicated in Table 1.

**Table 1:** Demographics of participants.

Demographics	Percent	Frequency (n)
Gender (n = 1811)		
Male	51.96%	941
Female	48.04%	870
Age (n = 1804)			
7–27 yr	2.22%	40
28–47 yr	12.30%	222
48–67 yr	58.15%	1049
68–97 yr	27.33%	493
Disability category (n = 1815)		
Visual	23.86%	433
Hearing	12.23%	222
Speaking	1.10%	20
Limbs	40.88%	742
Intelligence	9.31%	169
Mental	10.91%	198
Multiple	1.71%	31
Disability grade (n = 1785)		
I	15.52%	277
II	21.18%	378
III	25.21%	450
IV	38.09%	680
Education (n = 1815)			
Illiteracy	3.47%	63
Primary school	9.92%	180
Junior middle school	35.21%	639
Senior high school	37.68%	684
Junior college	9.75%	177
Undergraduate	3.97%	72
Current status (n = 1815)		
Worker	8.26%	150
Unemployment	11.41%	207
Student	0.61%	11
Retirement	79.72%	1447
Monthly income (n = 1815)		
<2000 yuan	15.98%	290
2000–3999 yuan	78.23%	1420
≥4000 yuan	5.79%	105
ADL (n = 1815)		
Normal	83.09%	1508
Impairment	16.91%	307
Hypertension (n = 1815)		
No	61.76%	1121
Yes	38.24%	694
Diabetes (n = 1815)		
No	84.74%	1538
Yes	15.26%	277
Hyperlipidemia (n = 1815)		
No	71.90%	1305
Yes	28.10%	510
Smoking		
No	No	1416
Yes	Yes	399

ADL = activities of daily living.

### 3.2 The depression status of the disabled

Based on the data obtained from our study, the average score of PHQ-9 was 4.46 ± 4.51. There were 1091 participants with no depressive symptoms (60.1%), 724 showed depressive symptoms, including 522 people with mild depressive symptoms (28.8%), 136 people with moderate depressive symptoms (7.5%), 48 respondents have moderate–severe depressive symptoms (2.6%), and 18 participants with severe depressive symptoms (1.0%). The incidence rate of depressive symptoms in the disabled people was 39.9%.

### 3.3 Single factor analysis of depression in people with disabilities

Table 2 shows the relationships between various factors and depression symptoms of the disabled persons. Based on the results, the incidence rate of depression between male and female was not statistically significant (*P* = .690). The detection rate of depression among different age groups was statistically significant (*P* < .01), the elderly group had the highest depression rate (42.7%), and the youth group had the lowest rate (28.0%). In addition, there was a statistically significant difference in the categories of disability, disability grade, and education degree (*P* < .05), while different monthly income and current working status had no influence on depressive symptoms.

**Table 2:** Single factor analysis of depression in people with disabilities.

		Depression		
Characteristics of study population	Frequency (n)	Positive	Negative	*χ*^2^ (*P*)
Gender				0.16 (.690)
Male	941	371	570	
Female	870	351	519	
Age (yr)				11.55 (.009)
7–27	40	15	25	
28–47	222	72	150	
48–67	1049	408	641	
68–97	493	223	270	
Disability category				15.33 (.018)
Visual	433	168	265	
Hearing	222	74	148	
Speaking	20	8	12	
Limbs	742	315	427	
Intelligence	169	53	116	
Mental	198	91	107	
Multiple	31	15	16	
Disability grade				9.23 (.026)
I	277	130	147	
II	378	148	230	
III	450	183	267	
IV	680	248	432	
Education				11.54 (.042)
Illiteracy	63	32	31	
Primary school	180	85	95	
Junior middle school	639	252	387	
Senior high school	684	258	426	
Junior college	177	63	114	
Undergraduate	72	34	38	
Current status				5.31 (.150)
Worker	150	50	100	
Unemployment	207	86	121	
Student	11	2	9	
Retirement	1447	586	861	
Monthly income (yuan)				0.58 (.750)
<2000	290	110	180	
2000–3999	1420	571	849	
≥4000	105	43	62	
ADL				98.30 (.000)
Normal	1508	524	984	
Impairment	307	200	107	
Hypertension				12.73 (.000)
No	1121	411	710	
Yes	694	313	381	
Diabetes				19.95 (.000)
No	1538	580	958	
Yes	277	144	133	
Hyperlipidemia				39.00 (.000)
No	1305	462	843	
Yes	510	262	248	
Smoking				0.314 (.575)
No	1416	560	856	
Yes	399	164	235	

ADL = activities of daily living.

According to the score of MBI, there were 307 people with activities of daily living disorder (16.9%). Among them, the incidence rate of depressive symptoms was 65.1%. While 1508 people can live completely self-independent (83.1%), and 34.7% depression detection rate. There was a statistical difference between the 2 depression incidence rates (*P* < .001). Besides, the rate of depression in the disabled with hypertension, diabetes, and hyperlipidemia was statistically significant (*P* < .001), and there was no statistical difference between smokers and the nonsmokers (*P* > .05).

### 3.4 Multifactor analysis of depression in people with disabilities

Selected the variables that are statistically significant in single factor analysis, and then analyzed by logistic regression analysis model (Table 3). The results showed that the influencing factors of depression in disabled persons are: grade I disability, intelligence disability, diabetes, hyperlipidemia, and impairment of ADL. By contrast to the other grade of disability, the risk of depression of grade I disableds was higher, OR = 1.37 (*P* < .05). Compared with the normal activities of daily living, the risk of depression of people with impairment activities of daily living was higher, OR = 3.23 (*P* < .001). Diabetes compared with non-diabetic the OR is 1.43 (*P* < .05). The people with hyperlipidemia may be more vulnerable to the depression, OR = 1.59 (*P* < .001). However, intelligence disability is a protective factor of depression (OR = 0.69, *P* < .05).

**Table 3:** Multifactor analysis of depression in people with disabilities.

			95% CI	
Variable	OR	*P*	Upper limit	Lower limit
Disability grade I	1.37	.022	1.05	1.79
Intelligence disability	0.69	.043	0.48	0.98
Diabetes	1.43	.014	1.08	1.90
Hyperlipidemia	1.59	.000	1.26	2.00
Impairment of ADL	3.23	.000	2.48	4.20
Constant	0.44	.000	–	–

ADL = activities of daily living.

## 4 Discussion

At present, most researches on disabled people mainly focus on improving their self-care ability of daily living, social support, family support, and physical rehabilitation in the world. Few studies focused on the mental health of the handicapped or only concerned with the mental state of disabled children and their guardians. The Beck Depression Scale, Patient's Health Questionnaire (PHQ-9), and Children's Depression Inventory (CDI) were used to assess the depression of the disabled children or their guardians, having reported the rate of depression is 19% to 47.5%.^[24–27]^ In our study, 262 subjects were younger than 48 years old, among which 87 people had depressive symptoms (33.2%). Therefore, we believe that the depressive symptoms of younger disabled people and their guardians are high detected. As a general practitioner, based on the community–family–individual model, we need to pay more attention to the psychological health of the young disabled family.

Nowadays, most studies on disabled people in China aim at improving their self-care ability of daily living, social support, family support, and physical rehabilitation. But the mental health of the disabled, especially the depression, is less focused and the assessment instruments are not uniform. A well-approved study showed that the Patient's Health Questionnaire (PHQ-9) had good reliability and validity in the measurement of depression, of which Cronbach's *α* = 0.89.^[28]^ PHQ-9 scale was used in this study and the depression detection rate of the disabled in the Jing’an district of Shanghai was 39.9%. Compared with healthy people, patients with chronic diseases, and even cancer patients, the incidence of depression among the disabled is higher, which deserves our attention. For example, Lu Ming et al's study reported that the incidence rate of depression in healthy people was 4.6%, while other studies reporting the people with hypertension and cancers were 23.7% and 32.8%, respectively.^[29–31]^

Somatic diseases have no significant effect on depression, but disability is a risk factor for depression, especially for disabled people over 60 years old.^[32]^ According to our study, the incidence rate of depression in 48 to 67 years and 68 to 97 years (38.9% and 45.2%) was higher than the younger age group (Table 2). As a special group, elderly people with history of chronic diseases such as hypertension, diabetes, and hyperlipidemia, have higher detection rate of depression, suggesting that we should pay more attention to the mental health of the elderly disabled persons, especially those with chronic diseases.^[33]^ However, there was no significant correlation between current working status and depression among people with disabilities. In our study, the incidence rate of depression between work and unemployment, work and retirement, unemployment, and retirement were not statistically significant (*P* > .05). The results tell us that focusing on increasing employment alone may not improve the depression of people with disabilities. Multifactor regression analysis (Table 3) showed that grade I disability, impairment activities of daily living, diabetes, and hyperlipidemia were associated with depression in the disabled. Among them, intelligence disability is a protective factor of depression, possibly because the emotional center of the person with intellectual disability is also incomplete.

The sample size of this study is large, including 1815 disabled participants, and it covers a wide range of people with different types of disabilities. We have explored the effects of activities of daily living, chronic diseases, and smoking on the depressive symptoms of the disabled in the community, which have certain innovation. Multifactor regression analysis showed that low level of culture, disability of self-care, the history of diabetes, and hyperlipidemia were possible risk factors of depression in people with disabilities. This suggests that it is of great significance to improve the self-care ability and control the chronic diseases of the disabled in the community. Prompting the health of the disabled in the community, and strengthening their chronic diseases management should be the general practitioners’ another significant and arduous task.

## 5 Limitations

This study is not without its limitations. The research of Wang et al showed that the utilization of health service resources was significantly correlated with mental health level.^[34]^ Research by Weich et al found that people who have no convenient transportation are at higher risk of depression than those who have better convenient transportation (OR = 1.84).^[35]^ What's more, the study found that physiological indicators such as physical pain were associated with depression in the physically handicapped.^[36]^ However, this article lacks data on health service utilization, transportation, and physiological indicators. In addition, our study is a cross-sectional study and cannot illuminate the exact causal relationship between the above-related factors and the depression. What's more, the study included a minority of people with impairment of activities of daily living (307), which may be related to the survey population. Most of the disabled people in the community are able to carry out daily life, while most of the disabled people who are unable to take care of themselves are in health centers, nursing homes, and even large hospitals, which we do not cover. But we believe that our study can provide clear clues and references for future studies and the results can be used to guide the community workers.

## 6 Conclusions

This study explored the prevalence and related factors of depression among people with disabilities in communities. Contents of the questionnaire include participants’ demographic characteristics, activities of daily living, chronic diseases, and mental health status, mainly using 2 scales, the MBI and the PHQ-9. As a general practitioner, it is of great significance to improve the self-care ability and manage the chronic diseases of the disabled in the community. The findings of this study provide clear clues and references for future mental health promotion/interventions with those disabled persons.

## Acknowledgments

Sincere thanks to all investigators and participants for their contributions in this research.

## Author contributions

**Conceptualization:** Limin Lao, Sunfang Jiang.

**Data curation:** Yahong Bi.

**Investigation:** Yahong Bi, Xincai Zhao, Yanyan Zhou.

**Methodology:** Yahong Bi, Limin Lao.

**Resources:** Limin Lao.

**Supervision:** Sunfang Jiang.

**Writing – original draft:** Yahong Bi.

**Writing – review & editing:** Sunfang Jiang, Yahong Bi, Xincai Zhao.
